# Carbon disulfide exposure estimate and prevalence of chronic diseases after carbon disulfide poisoning-related occupational diseases

**DOI:** 10.1186/s40557-017-0208-6

**Published:** 2017-10-26

**Authors:** Hweemin Chung, Kanwoo Youn, Kyuyeon Kim, Kyunggeun Park

**Affiliations:** 1 Department of Occupational & Environmental Medicine, Wonjin Green Hospital (Seoul), Sagajeong-ro 49-gil 53, Jungrang-gu, Seoul, South Korea; 2 Department of Internal Medicine, Wonjin Green Hospital (Guri), Dongguneung-ro 65-gil, Guri Si, Gyeonggi-do, South Korea

**Keywords:** Carbon disulfide poisoning, Rayon-manufacturing plant, Ex-workers, Cumulative exposure estimate

## Abstract

**Background:**

In Korea, Carbon disulfide (CS_2_) toxicity was an important social problem from the late 1980s to the early 1990s but there have been few large-scale studies examining the prevalence of diseases after CS_2_ exposure discontinuance. So we investigated past working exposure to CS_2_ characteristics from surviving ex-workers of a rayon manufacturing plant including cumulative CS_2_ exposure index. Furthermore, we studied the prevalence of their chronic diseases recently after many years.

**Methods:**

We interviewed 633 ex-workers identified as CS_2_ poisoning-related occupational diseases to determine demographic and occupational characteristics and reviewed their medical records. The work environment measurement data from 1992 was used as a reference. Based on the interviews and foreign measurement documents, weights were assigned to the reference concentrations followed by calculation of individual exposure index, the sum of the portion of each time period multiplied by the concentrations of CS_2_ during that period.

**Results:**

The cumulative exposure index was 128.2 ppm on average. Workers from the spinning, electrical equipment repair, and motor repair departments were exposed to high concentrations of ≥10 ppm. Workers from the maintenance of the ejector, manufacturing of CS_2,_ post-process, refining, maintenance and manufacturing of viscose departments were exposed to low concentrations below 10 ppm. The prevalence for hypertension, coronary artery disease, cerebrovascular disease, diabetes, arrhythmia, psychoneurotic disorder, disorders of the nervous system and sensory organ were 69.2%, 13.9%, 24.8%, 24.5%, 1.3%, 65.7%, 72.4% respectively.

**Conclusions:**

We estimated the individual cumulative CS_2_ exposure based on interviews and foreign measurement documents, and work environment measurement data. Comparing the work environment measurement data from 1992, these values were similar to them. After identified as CS_2_ poisoning, there are subjects over 70 years of average age with disorders of the nervous system and sensory organs, hypertension, psychoneurotic disorder, cerebrovascular disease, diabetes, coronary artery disease, and arrhythmia. Because among ex-workers of the rayon manufacturing plant, only 633 survivors recognized as CS_2_ poisoning were studied, the others not identified as CS_2_ poisoning should also be investigated in the future.

## Background

Carbon disulfide (CS_2_) is a substance in which two sulfur atoms are bonded to one carbon molecule. It is colorless and has high volatility and flammability. CS_2_ has been an important industrial chemical since the 1800s because of its many useful properties, including its ability to solubilize fats, rubbers, phosphorus, sulfur, and other elements. In the early 1900s, CS_2_ was used in large quantities during the synthesis of viscose rayon. CS_2_ is absorbed mainly by inhalation of steam whereupon it enters the bloodstream and is distributed throughout the body. CS_2_ can also be absorbed via other mucous membranes such as those of the eyes and skin. The target organs include the central nervous system, cardiovascular system, peripheral nervous system, reproductive system, eyes, and skin [1–3].

Among the health effects of CS_2_, the most commonly known is the increase in the incidence of cerebral cardiovascular diseases due to sclerotic changes in blood vessels cause by impaired lipid metabolism. One of the specific vascular effects of CS_2_ is the development of retinal lesions characterized by petechiae of the retina [4] or microalbuminuria. Cerebral cardiovascular disease as well as central nervous system disorders are among the major health effects of CS_2_ poisoning. In addition, polyneuropathy may occur after chronic exposure to low doses [5]. Parkinson’s symptoms such as cogwheel rigidity, resting tremor, action tremor, bradykinesia, cerebellar ataxia, and vertebral symptoms may occur [6]. Diffuse encephalopathy has been observed in clinical studies, psychiatric studies, and neuroradiologic studies for decades [7]. In addition, reports claim that CS_2_ exposure is associated with the development of endocrine disease associated with impaired lipid metabolism. Also, owing to its effects on mental health, CS_2_ poisoning results in excessive nervousness, uncontrolled anger, rapid mood changes, paranoia, and suicidal tendencies [8].

Thus, CS_2_ poisoning affects the cardiovascular system [9–11], the cerebrovascular system [12–14], the endocrine system [15, 16], mental health [8], and the nervous system [5–7, 15].

In Korea, CS_2_ toxicity was an important social problem from the late 1980s to the early 1990s, but paradoxically, it contributed to the development of the industrial safety and health system in Korea. Wonjin Rayon Co., Ltd. imported mechanical equipment from Japan, and since 1962 has manufactured viscose rayon and used CS_2_ as the main raw material in the process. In 1966 about 15 tons of viscose rayon was produced per day, which increased to 50 tons per day in 1976. Since then, a deficit has accumulated owing to changes in the industrial environment, and CS_2_ toxicity became a social problem. Wonjin Rayon Co., Ltd. was closed in 1993. Until the mid-1970s when production was high, about 3000 employees worked in the manufacturing plant annually, but the number of employees at the time of the closure was about 1000.

The cases of CS_2_ poisoning of Wonjin Rayon Co., Ltd. were reported around 1988. Accordingly, the Graduate School of Public Health of Seoul National University carried out an epidemiological survey in 1992, and since then, the number of patients with CS_2_ poisoning has increased drastically. The procedure for recognizing occupational diseases was as follows. As a first step, a person with a career in carbon disulfide working at Wonjin Rayon confirmed his working career at the Wonjin workers’ occupational medicine committee and completed a medical treatment application. As a second step, he examined a primary care from Seongsoo’s clinic and received a medical report on CS_2_ poisoning. As a third step, he was examined at a university hospital. As a final step, he was judged to have an occupational disease according to the standard of occupational accidents recognized by the Ministry of Labor (Industrial Accident Compensation Insurance Act, Article 39, Ministry of Labor). In this way, a total of 910 persons were recognized as CS_2_ poisoning-related occupational diseases, among whom 208 persons died and 702 persons survived by November 2016.

However, despite the number of studies reporting CS_2_ poisoning in Korea, the social significance of this issue was underreported. In addition to the epidemiologic survey [16], a few case reports and studies on the clinical symptoms and distribution of a single disease among a small number of workers exposed to CS_2_ have been reported [7, 17]. There have been few large-scale studies examining the prevalence of diseases after CS_2_ exposure discontinuance.

The purpose of this study was to investigate past working exposure to CS_2_ characteristics from investigable 633 ex-workers identified as CS_2_ poisoning-related occupational diseases including cumulative CS_2_ exposure index. Furthermore, we studied the prevalence of their major chronic diseases associated with CS_2_ poisoning recently after many years. We approached ex-workers of Wonjin Rayon Co., Ltd., who were identified as CS_2_ poisoning-related occupational diseases, via the Wonjin Industrial Accident Society.

## Methods

### Study population

From 1962 to 1993, there were about 12,000 employees at Wonjin Rayon Co., Ltd., but all of their information is not available. Only 910 persons were recognized as having occupational diseases. The standard of occupational accidents about CS_2_ poisoning or its secondary diseases is shown in Table 1. Initially, the approval name of industrial accidents was carbon disulfide poisoning, and further details are not available. Of these, 208 persons died and 702 persons survived until November 2016. Finally, 633 persons, excluding 69 who refused to participate in the survey or were unable to express their opinions, were selected for the study. This study was approved by the ethics reviews board of Wonjin Institute for Occupational and Environmental Health.

**Table 1:** Standards of occupational accidents about CS_2_ poisoning or its secondary diseases^a^

20. CS_2_ poisoning or its secondary diseases
Ga. A worker who has worked for more than two years on work exposed to CS_2_ vapor of around 10 ppm shall be considered as a work-related disease if any of the following symptoms or symptoms appear.
(1) When there is one of retinal microvessels, multiple cerebral infarction, capillary glomerulosclerosis on renal biopsy. However, it excludes diseases caused by causes other than CS_2_, such as diabetes, hypertension and vascular disorders.
(2) Retinal lesions other than microvascular lesions, multiple peripheral nerve lesions, optic neuritis, coronary heart disease, central nervous system disorders or psychiatric disorders. However, it excludes diseases caused by causes other than CS_2_, such as diabetes, hypertension and vascular disorders.
(3) There is one of (2) and there is at least one of kidney disorder, liver disorder, hematopoietic system disorder, reproductive system disorder, sensory nerve impairment disorder, and hypertension.
Na. A worker who is exposed to more than 20 ppm of CS_2_ vapor for more than two weeks shall be considered as a work-related disease if they present with sudden manifestations of mental disorders such as confusion, delirium, schizophrenia and bipolar disorder.
Da. When exposed to a large or high concentration of CS_2_ vapor, symptoms of acute intoxication such as consciousness disorder are regarded as occupational diseases.

^a^Industrial Accident Compensation Insurance Act, Article 39, Ministry of Labor, 1997

Through the medical records of the 6 hospitals that manage the health of the subjects, the results of the medical examinations conducted over the past 1 year, prescriptions, and clinical history were reviewed. The name of the disease listed in data for the 295 diseases classified by the Health Insurance Statistical Yearbook of 2015 is composed of several diagnostic codes corresponding to the International Classification of Disease (ICD) codes as shown in Table 2. In addition, the Wonjin Industrial Accident Society, for 2 months since September 19, 2016, individually contacted the research subjects and conducted a one-on-one interview using a questionnaire to determine working history, demographic characteristics, health behavior, and habits. Information regarding working history included items such as year of employment, department of work, contents of work, move within/across departments, and period of work.

**Table 2:** ICD-10 code of target disease

Disease	Classification of 298 Diseases^a^	ICD-10 code^b^	
Hypertension			
	Essential(primary) hypertention Other hypertensive diseases	I10	Essential (primary) hypertension
		I11	Hypertensive heart disease
		I12	Hypertensive renal disease
		I13	Hypertensive heart and renal disease
		I15	Secondary hypertension
Coronary artery disease			
	Acute myocardial infarction Other ischaemic heart diseases	I20	Angina pectoris
		I21	Acute myocardial infarction
		I22	Subsequent myocardial infarction
		I23	Certain current complications following acute myocardial infarction
		I24	Other acute ischaemic heart diseases
		I25	Chronic ischemic heart disease
Cerebrovascular disease			
	Transient cerebral ischaemic attacks and related syndromes Cerebral palsy and other paralytic syndromes	G45	Transient cerebral ischaemic attacks and related syndromes
		G50	Disorders of trigeminal nerve
		G51	Facial nerve disorders
		G52	Disorders of other cranial nerves
		G53	Cranial nerve disorders in diseases classified elsewhere
		G80	Cerebral palsy
		G81	Hemiplegia
		G82	Paraplegia and tetraplegia
		G83	Other paralytic syndromes
Diabetes mellitus			
	Diabetes mellitus	E10	Type 1 diabetes mellitus
		E11	Type 2 diabetes mellitus
		E12	Malnutrition-related diabetes mellitus
		E13	Other specified diabetes mellitus
		E14	Unspecified diabetes mellitus
Arrhythmia			
	Conduction disorders and cardiac arrhythmias	I44	Atrioventricular and left bundle-branch block
		I45	Other conduction disorders
		I46	Cardiac arrest
		I47	Paroxysmal tachycardia
		I48	Atrial fibrillation and flutter
		I49	Other cardiac arrhythmias
Psychoneurotic disorder			
	Schizophrenia schizotypal and delusional disorders Mood [affective] disorders Neurotic,stress-related and somatoform disorders	F20	Schizophrenia
		F21	Schizotypal disorder
		F22	Persistent delusional disorders
		F23	Acute and transient psychotic disorders
		F24	Induced delusional disorder
		F25	Schizoaffective disorders
		F28	Other nonorganic psychotic disorders
		F29	Unspecified nonorganic psychosis
		F40	Phobic anxiety disorders
		F41	Other anxiety disorders
		F42	Obsessive-compulsive disorder
		F43	Reaction to severe stress, and adjustment disorders
		F44	Dissociative [conversion] disorders
		F45	Somatoform disorders
		F48	Other neurotic disorders
Disorders of the nervous system and sensory organs			
	Nerve, nerve root and plexus disorders Other diseases of the nervous system	G12	Spinal muscular atrophy and related syndromes
		G13	Systemic atrophies primarily affecting central nervous system in diseases classified elsewhere
		G21	Secondary parkinsonism
		G22	Parkinsonism in diseases classified elsewhere
		G24	Dystonia
		G25	Other extrapyramidal and movement disorders
		G26	Extrapyramidal and movement disorders in diseases classified elsewhere
		G37	Other demyelinating diseases of central nervous system
		G56	Mononeuropathies of the upper limb
		G57	Mononeuropathies of the lower limb
		G58	Other mononeuropathies
		G59	Mononeuropathy in diseases classified elsewhere
		G62	Other polyneuropathies
		G64	Other disorders of the peripheral nervous system
		G70	Myasthenia gravis and other myoneural disorders
		G72	Other myopathies
		G73	Disorders of the myoneural junction and muscle in diseases classified elsewhere
		G90	Disorders of the autonomic nervous system
		G92	Toxic encephalopathy
		G93	Other disorders of the brain
		G94	Other disorders of the brain in diseases classified elsewhere
		G95	Other diseases of the spinal cord
		G96	Other disorders of the central nervous system
		G98	Other disorders of the nervous system not elsewhere classified
		G99	Other disorders of the nervous system in diseases classified elsewhere

^a^2015 National Health Insurance Statistical Yearbook

^b^International Classification of Disease-10

### Estimation of CS_2_ exposure

To estimate past exposure to CS_2_ without actual measurement data, the work environment measurement data from 1992 used by the Seoul National University Graduate School of Public Health [18] were analyzed by 15 departments and used as the reference concentration. Based on the interviews of the study subjects, facility-related weights were assigned to the baseline concentrations according to the change in the equipment repair time and production amount. Exposure index in the absence of measurement data were weighted with reference to foreign measurement documents.

#### CS_2_ exposure by job department

The results of the Ministry of Labor’s environment survey of Wonjin Rayon Co., Ltd. conducted in 1991 and the average of the work environment measurement data estimated by the Seoul National University Graduate School of Public Health in 1992 were applied as standard concentrations. The Maintenance Department, Chemical Engineering Department, and General Affairs Department, which do not have measurement data, applied estimates by referring to the contents of the work. Table 3 shows the results of the workers’ working areas and the environmental characteristics of CS_2_ exposure (Table 3). Based on studies conducted in the EU a time-weighted average (TWA) of 5 ppm for departments exposed to CS_2_ concentrations of ≥5 ppm was classified as high exposure. Based on studies conducted in the National Institute for Occupational Safety and Health a TWA of 1 ppm for departments exposed to CS_2_ concentrations of <1 ppm was classified as low exposure. Departments exposed to concentrations of ≥1 ppm and <5 ppm were classified as involving moderate exposure.

**Table 3:** Establishing criteria for CS_2_ exposure concentration by department

Department	CS_2_ -exposed Concentration (ppm)	Job Contents
High exposure concentration department		
Spinning		
Doping	6.53	Threading up a spinning machinery and taking out the cake, As CS_2_ is released during rayon regeneration, exposure concentration is highest with direct exposure.
Circuit	6.58	In the spinning room having a tangled or broken thread, As CS_2_ is released during rayon regeneration, exposure concentration is highest with direct exposure.
Motor Repair	3.79	Repairing motors of a spinning machinery
Moderate exposure concentration department		
Electrical Equipment Repair	4.33	High amount of spinning room work while repairing electrical equipment due to mechanical corrosion problems.
Maitenance of Ejector	3.03	It is intermittent for reasons such as machine maintenance, but working within the spinning department
Collection of Sulfuric acid	3.79	CS_2_ mixed in the recovered solution.
Manufacturing of CS_2_	2.77	Exposure to high concentrations of CS_2_ for a short time while manufacturing, but protective gear is used.
Post-Process		
Packing	3.6	Restricted to the spinning room
Carrying	1.96	Working in a spinning room for a long time to transport rayon.
Refining	2	Exposure to remaining CS_2_ while processing semi-finished cake
Maintenance	2.16	Estimated at 50% of that in the electrical equipment repair department
Manufacturing of Viscose	1.35	
Low exposure concentration department		
Water Treatment	0.45	Sewage inspection and cleaning in the spinning room
Manufacturing of Chemicals	0.23	Estimated at 50% of that in the water treatment department, production of caustic soda and liquid chlorine, lower possibility of direct exposure.
General Affairs	0.12	Estimated at 50% of that in the manufacturing department

#### Weights by work period

Through interviews of 30 workers conducted to identify objective facts about past CS_2_ exposure and the 1991 Wonjin Rayon Co., Ltd. Status report, the cost of investing in work environment management was found to gradually increase over time. By taking into account the average concentration for each period identified in foreign literature and the changes in the work environment, facilities, and production volume of Wonjin Rayon Co., Ltd., we divided work period into three stages and weighed them according to the timing (Table 4).

**Table 4:** Setting weight by operating period

Operating Period (year)	Weight by period	Evidence
1966 (Initial operation)-1974	3.5	The average concentration of CS_2_ in the spinning division in the 1960s (20 ppm) was about 3.5 times the average concentration of the total spinning division measured in 1991 (5.6 ppm).
1975–1989	2.75	The average concentration in the spinning division measured in the 70s and 80s (10–15 ppm) was about 2.25 times the average concentration in the total spinning division measured in 1991 (5.6 ppm). Since 1975, the production of rayon has increased. According to the operator interview, the exhaust system has changed, and the screen breaks down due to the age of the equipment. Thus, an additional weight of 0.5 was included.
1990–1993 (Upon closure)	0.5	The compliance towards wearing personal protective gear was relatively good. The highest CS_2_ exposure concentration measured in protective equipment (3.2 ppm) was about 0.5 times that measured in 1991 (5.6 ppm).

In 1973, the National Institute for Occupational Safety and Health in the US showed that the 8-h TWA of airborne exposure in the workplace, such as during spinning and cutting in the rayon plant, exceeded 200 ppm in 12 of 36 cases; in 7 cases, the 8-h TWA exceeded 100 ppm. In another factory, in more than 50% of the 196 cases analyzed, the concentration of CS_2_ exceeded 100 ppm, and the time weighted average was 11.2 ppm (range: 0.9–127 ppm) [19]. Analysis of the measurement data of a rayon factory in Finland from 1945 to 1967 showed that the concentration of CS_2_ was generally estimated to be more than >40 ppm before 1950, 20–40 ppm between 1950 and 1960, and 10–30 ppm in the 1960s [20]. The average concentration of CS_2_ in the spinning division in the 1960s (20 ppm), which was identified in previous foreign literature, was about 3.5 times the average concentration of the total spinning division measured in 1991 (5.6 ppm). As a result, the weight of the period from early 1966 to 1974 was 3.5. The average concentration in the spinning division measured in the 70s and 80s (10–15 ppm) was about 2.25 times the average concentration in the total spinning division measured in 1991 (5.6 ppm).The weight of the period from 1975 to 1989 was estimated as 2.75 by adding 0.5 in consideration of the working environment at that time. From 1990 to 1993, the compliance towards wearing personal protective gear was relatively good and the highest CS_2_ concentration in the protective area was 3.2 ppm. Since the concentration was 0.5 times the reference concentration, the weight assigned was 0.5.

Based on these data, the individual cumulative exposure index and the individual average exposure concentration were calculated as shown in Table 5.

**Table 5:** Calculation of the cumulative exposure index and time-weighted average concentration of CS_2_

• cumulative exposure index (CEI) = Σ (working years x reference concentration x weight)	
• time weighted average CS_2_ concentration = Σ (the hours in the workday x reference concentration x weight) / the hours in the workday of exposure to CS_2_	

## Results

### Characteristics of the study population

Characteristics of the workers included in this study are shown in Table 6. The subjects of this study were mainly male, accounting for 86.3% of the population; the mean age was 70.7 years (70.7 ± 7.5 years) and the mean age at the time of study entry was 27.9 years (27.9 ± 5.3 years). Of the subjects, 41.1% consumed alcohol more than once a week and 60.8% were current smokers.

**Table 6:** Demographic and employment characteristics of the study subjects

		Number of the subjects		Mean	SD
		N	%		
Demographic characteristics					
Total		633	100.0		
Sex	Male	546	86.3		
	Female	87	13.7		
Age distribution (year)	50–59	57	52.6		
	60–69	196	69.4		
	≥ 70	380	71.6		
Smoking	No	212	33.5		
	Yes	385	60.8		
	No answer	36	5.7		
Alcohol consumption (days/week)	<1	336	53.1		
	≥1	260	41.1		
	No answer	37	5.9		
Employment characteristics					
Age at first employment (years)				27.9	5.3
Age at date last observed among survivors (years)				70.7	7.5
Year in the first employment (year)				1974.2	6.2
	<1975	339	53.6		
	1975–1993	294	46.4		
Duration of employment (years)				12.8	5.6
	<10	204	29.7		
	10–<20	379	59.9		
	≥20	66	10.4		
Cumulative CS_2_ exposure index (ppm·yr)				128.2	82.2
	0–<67^a^	169	26.7		
	67–<134	206	32.5		
	≥134^b^	258	40.8		
Time-weighted average CS_2_ concentration (ppm)				10.1	4.9
	0- < 5^c^	112	17.7		
	5–<10^d^	199	31.4		
	≥10	322	50.9		
Department distribution by CS_2_ exposure concentration					
	Low (<5 ppm)^e^	340	53.7		
	High (≥5 ppm)	293	46.3		

^a^lower one third defined by distribution of metric among all the study subject

^b^upper one third defined by distribution of metric among all the study subject

^c^EU - Commission Directive 2006/15/EC of 7 February 2006 establishing a second list of indicative occupational exposure limit values

^d^Industrial Safety and Health Act enforcement regulations separate Table 3 of 11 Amendment of allowable standard of exposure concentration by harmful factor, IRE - 2010 Code of Practice for the Safety, Health and Welfare at Work (Chemical Agents) Regulations 2001. Published by the Health and Safety Authority

^e^EU - Commission Directive 2006/15/EC of 7 February 2006 establishing a second list of indicative occupational exposure limit values

### CS_2_ exposure

According to working department, 53.7% of the subjects were working in departments with low exposure, with a TWA CS_2_ concentration of <5 ppm, and 46.3% of subjects were in departments with high exposure, with a TWA CS_2_ concentration of ≥5 ppm. The average employment duration was 12.8 years (12.8 ± 5.6 years). To estimate the cumulative CS_2_ exposure index, we calculated the cumulative exposure index as 128.2 ppm (128.2 ± 82.2 ppm) considering all the departments they moved to. The TWA concentration was 10.1 ppm (10.1 ± 4.9 ppm) (Table 6). Furthermore, 93.2%, 76.9%, and 61.5% of workers from the spinning, electrical equipment repair, and motor repair departments, respectively, were exposed to high concentrations of ≥10 ppm on average. During the maintenance of the ejector, manufacturing of CS_2,_ post-process, refining, maintenance and manufacturing of viscose departments, 80.0%, 91.7%, 99.3%, 100.0%, 100.0% and 98.2% workers, respectively, were exposed to concentrations below 10 ppm on average. These values are similar to those reported in the 1991–1992 work environment measurement data (Table 7).

**Table 7:** CS_2_ exposure concentrations by department

		Workers Exposed to CS_2_ (%)											
		Department (*N* = 633**)**											
		Spinning	Electrical Equipment Repair	Motor Repair	Collection of Sulfuric acid	Maintenance of ejector	Manufacturing of CS_2_	Post-Process	Refining	Maintenance	Manufacturing of Viscose	Water Treatment	General Affairs
CS_2_ exposure concentration^a^ (ppm)													
0- < 5^b^	N	10	1	0	0	1	0	37	2	9	52	0	0
	%	3.6	7.7	0.0	0.0	2.5	0.0	25.5	25.0	56.3	96.3	0.0	0.0
5- < 10^c^	N	9	2	10	12	31	11	107	6	7	1	2	1
	%	3.2	15.4	38.5	34.3	77.5	91.7	73.8	75.0	43.7	1.9	100.0	50.0
≥10	N	261	10	16	23	8	1	1	0	0	1	0	1
	%	93.2	76.9	61.5	65.7	20.0	8.3	0.7	0.0	0.0	1.8	0.0	50.0
total (N)		280	13	26	35	40	12	145	8	16	54	2	2

^a^Time-weighted average CS_2_ concentration

^b^EU - Commission Directive 2006/15/EC of 7 February 2006 establishing a second list of indicative occupational exposure limit values

^c^Industrial Safety and Health Act enforcement regulations separate Table 3 of 11 Amendment of allowable standard of exposure concentration by harmful factor, IRE - 2010 Code of Practice for the Safety, Health and Welfare at Work (Chemical Agents) Regulations 2001. Published by the Health and Safety Authority

### The prevalence of chronic diseases for the subjects

Table 8 presents the prevalence of diseases for subjects [21]. The prevalence for hypertension, coronary artery disease, cerebrovascular disease, diabetes, arrhythmia, psychoneurotic disorder, disorders of the nervous system and sensory organ were 69.2%, 13.9%, 24.8%, 24.5%, 1.3%, 65.7%, 72.4% respectively.

**Table 8:** Disease prevalence for the study subjects

		Observed Events	Study population	Disease Prevalence
		N	N	%
Disease				
Hypertension		438	633	69.2
Coronary artery disease		88	633	13.9
Cerebrovascular disease		157	633	24.8
Diabetes mellitus		155	633	24.5
Arrhythmia		8	633	1.3
Psychoneurotic disorder		416	633	65.7
Disorders of the nervous system and sensory organs		458	633	72.4

## Discussion

We investigated past working exposure to CS_2_ characteristics from investigable 633 ex-workers identified as CS_2_ poisoning-related occupational diseases including cumulative CS_2_ exposure index. Furthermore, we studied the prevalence of their major chronic diseases associated with CS_2_ poisoning recently after many years. Comparing work environment measurement data reported in the 1991–1992, these values were similar to them. After the subjects were identified as CS_2_ poisoning-related occupational diseases, there are subjects over 70 years of average age with chronic diseases. Disorders of the nervous system and sensory organs, hypertension, psychoneurotic disorder, cerebrovascular disease, diabetes, coronary artery disease, and arrhythmia are the descending order of the prevalence for their chronic diseases.

In our study, there were several advantages to the survey method. We used an evidence-based approach to estimate past exposure to CS_2_ and to examine the presence or absence of chronic diseases. In the study of Nishiwaki et al. [22], 432 male workers exposed to CS_2_ and 402 male referent workers in 11 Japanese viscose rayon factories were studied at baseline; 750 of these were followed up. Brain magnetic resonance imaging was performed at both baseline and follow-up surveys. They found that the CS_2_ concentrations in the workers’ breathing zones and the level of 2-thiothiazolidine-4-carboxylic acid (TTCA), a metabolite of CS_2_, were measured twice a year since the spring of 1993 during the study period. Individual exposure level was represented by the arithmetic mean of the concentrations of TTCA and CS_2_ for 6 years. Similarly, our study also used the TWA concentration in the workers’ breathing zone as a means of assessing CS_2_ exposure. However, the data could be used only to estimate past exposures. On the other hand, restriction of TTCA estimation to urine samples as a means of biomonitoring could be a limitation. Kotseva et al. [23] also conducted a similar CS_2_ exposure assessment method. Concentrations of CS_2_ were assessed using stationary measurements and personal sampling methods. A preliminary estimate of CS_2_ concentrations in the manufacturing area was achieved by short-term sampling. To estimate the CS_2_ exposure at the time of the study, 8-h weighted average personal breathing zone samples were collected from some workers in each job category. A cumulative exposure index was calculated for each worker by multiplying the number of years he had held a particular job with the CS_2_ concentrations in that job. According to the degree of personal exposure, the study population was allocated to three groups: highly exposed, moderately exposed, and non-exposed.

In contrast with our approach (review of prescription and medical records and medical examination results), Nishiwaki et al. [22] used the medical records of the company via interview when examining the medical history of the subjects. Thus, our study has an advantage in that the prevalence estimated is more accurate [15, 23–26], because it relies on past history by self-filling or one-on-one interview questionnaires.

To estimate CS_2_ exposure index prior to 1986, exposure index was estimated by applying baseline and weights according to work period in 1991 and 1992, where the data were available. Because the departmental exposure index recorded in existing work environment measurement results (Table 3) and that of the present study (Table 7) showed a similar distribution, the exposure assessment can be deemed appropriate. There was an exception to this distribution. For example, the department with high CS_2_ exposure contained workers with moderate or low exposure because of the short working period in the high-exposure department since the 1990s. The presence of workers with high CS_2_ exposure in the department with low exposure indicates that these workers in the production department moved to the low-exposure department.

The average age of the subjects was 70.7, and most diseases that they have are chronic. The prevalence for hypertension, coronary artery disease, cerebrovascular disease, diabetes, arrhythmia, psychoneurotic disorder, disorders of the nervous system and sensory organ were 69.2%, 13.9%, 24.8%, 24.5%, 1.3%, 65.7%, 72.4% respectively. Vanhoorne et al. showed significant effects of the CS_2_ cumulative exposure index on systolic BP, diastolic BP [11]. Hyung-Joon Jhun et al. suggested that CS_2_ poisoned subjects have an increased risk of ECG abnormalities even after the exposure to CS_2_ is no longer present [27]. Several cross-sectional epidemiological studies have demonstrated that exposure to CS_2_ can result in increased cardiovascular risk factors and thus mortality [9–11, 28]. Recently, long-term exposure to CS_2_ was found to be an independent risk factor for changes in intima-media thickness in a German rayon manufacturing plant [9]. Long-term CS_2_ exposure has also been associated with increased cardiovascular mortality [11]. The abnormalities of cerebrovascular system were evaluated in CS_2_ poisoning cases [12–14]. Eunil Lee et al. [12] suggested that CS_2_ exposure could lead to a decrease of cerebral vasoreactivities by the atherosclerotic change of cerebral vessels. In a study of the health effects of CS_2_ on metabolic syndrome, the CS_2_-poisoned group showed an increased risk for abdominal obesity and high fasting glucose levels after adjusting for covariates [16]. Krstev et al. [29] reported neuropsychiatric effects in workers with occupational exposure to CS_2_. In a study of the effects of CS_2_ on the peripheral nervous system using nervous conduction, the number of cases with abnormal nerve conduction results increased according to age and duration of exposure [5]. Also in this study, after ex-workers of the rayon manufacturing plant were identified as CS_2_ poisoning-related occupational diseases, there are subjects with chronic diseases as mentioned earlier. Disorders of the nervous system and sensory organs, hypertension, psychoneurotic disorder, cerebrovascular disease, diabetes, coronary artery disease, and arrhythmia are the descending order of the prevalence for their chronic diseases.

This study has the following limitations. First, concurrent exposure to other chemicals as well as failure to control for other disease risk factors, such as aging, is a limitation of this study. Second, diseases involving high mortality rates and severity, such as cerebrovascular diseases, involve a relatively large number of deaths and intensive care unit admission, making follow-up observations difficult. Third, a prevalence-incidence bias could occur [30]. Fourth, because demographic and work-related factors were investigated using a one-on-one interview survey approach, the intention of researchers and responders could be reflected. Fifth, the research subjects were very selective and could not be compared with the general population. Finally, there is a problem with the reliability of the reference data, and it is impossible to calculate the accurate exposure amount according to the change in the work environment during the period. The date regarding the working environment at Wonjin Rayon Co., Ltd. and medical examination results prior to 1986 were not available. There is no measurement data except for the very limited measurement results from 1986 to 1990, and these results also have many limitations in terms of the reliability of the results owing to the lack of samples, lack of cooperation of the company, and method of analysis. In order to overcome these limitations, exposure index was estimated by applying periodic weights with reference to foreign documents.

Because among ex-workers of the rayon manufacturing plant, only 633 survivors recognized as CS_2_ poisoning-related occupational were studied, the others not identified as CS_2_ poisoning occupational diseases should also be investigated. A patient-control study will be needed in the future.

## Conclusions

We investigated past working exposure to CS_2_ characteristics from investigable 633 ex-workers of the rayon manufacturing plant who were identified as CS_2_ poisoning-related occupational diseases including cumulative CS_2_ exposure index based on the interviews, foreign measurement documents, and work environment measurement data reported in the 1991–1992. Comparing work environment measurement data reported in the 1991–1992, these values were similar to them. It is known that CS_2_ poisoning affects the cardiovascular system, the cerebrovascular system, the endocrine system, mental health, and the nervous system. Thus, we studied the prevalence of their major chronic diseases associated with CS_2_ poisoning recently after many years. Also in this study, after the subjects were identified as CS_2_ poisoning-related occupational diseases, there are subjects over 70 years of average age with disorders of the nervous system and sensory organs, hypertension, psychoneurotic disorder, cerebrovascular disease, diabetes, coronary artery disease, and arrhythmia. Because among ex-workers of the rayon manufacturing plant, only 633 survivors recognized as CS_2_ poisoning-related occupational were studied, the others not identified as CS_2_ poisoning occupational diseases should also be investigated. A patient-control study will be needed in the future.

## Abbreviations

CI: Confidence interval
CS_2_: Carbon disulfide
ICD: International classification of disease
OR: Odds ratio
SPR: Standardized prevalence ratio
TTCA: 2-thiothiazolidine-4-carboxylic acid
TWA: Time weighted average
